# Gliadin proteolytical resistant peptides: the interplay between structure and self-assembly in gluten-related disorders

**DOI:** 10.1007/s12551-021-00856-z

**Published:** 2021-11-18

**Authors:** Maria Georgina Herrera, Veronica Isabel Dodero

**Affiliations:** 1 Department of Physiology and Molecular and Cellular Biology, Institute of Biosciences, Biotechnology and Translational Biology (iB3), Faculty of Exact and Natural Sciences, University of Buenos Aires, Buenos Aires C1428EG, Argentina; 2 Institute of Biochemistry and Pathobiochemistry, Ruhr-University Bochum, 44801 Bochum, Germany; 3 Organic and Bioorganic Chemistry, Department of Chemistry, Bielefeld University, 33615 Bielefeld, Germany

**Keywords:** Gluten-related disorders, Self-assembly, Spectroscopic methods, Microscopies, Secondary structure

## Abstract

In recent years, the evaluation of the structural properties of food has become of crucial importance in the understanding of food-related disorders. One of the most exciting systems is gliadin, a protein in wheat gluten, that plays a protagonist role in gluten-related disorders with a worldwide prevalence of 5%, including autoimmune celiac disease (CeD) (1%) and non-celiac wheat sensitivity (0.5–13%). It is accepted that gliadin is not fully digested by humans, producing large peptides that reach the gut mucosa. The gliadin peptides cross the lamina propria eliciting different immune responses in susceptible patients. Many clinical and biomedical efforts aim to diagnose and understand gluten-related disorders; meanwhile, the early stages of the inflammatory events remain elusive. Interestingly, although the primary sequence of many gliadin peptides is well known, it was only recently revealed the self-assembly capability of two pathogenic gliadin fragments and their connection to the early stage of diseases. This review is dedicated to the most relevant biophysical characterization of the complex gliadin digest and the two most studied gliadin fragments, the immunodominant 33-mer peptide and the toxic p31-43 in connection with inflammation and innate immune response. Here, we want to emphasize that combining different biophysical methods with cellular and in vivo models is of key importance to get an integrative understanding of a complex biological problem, as discussed here.

## Gliadin and its proteolytical resistant peptides self-assemble under biologically relevant conditions

**Fig. 1:**
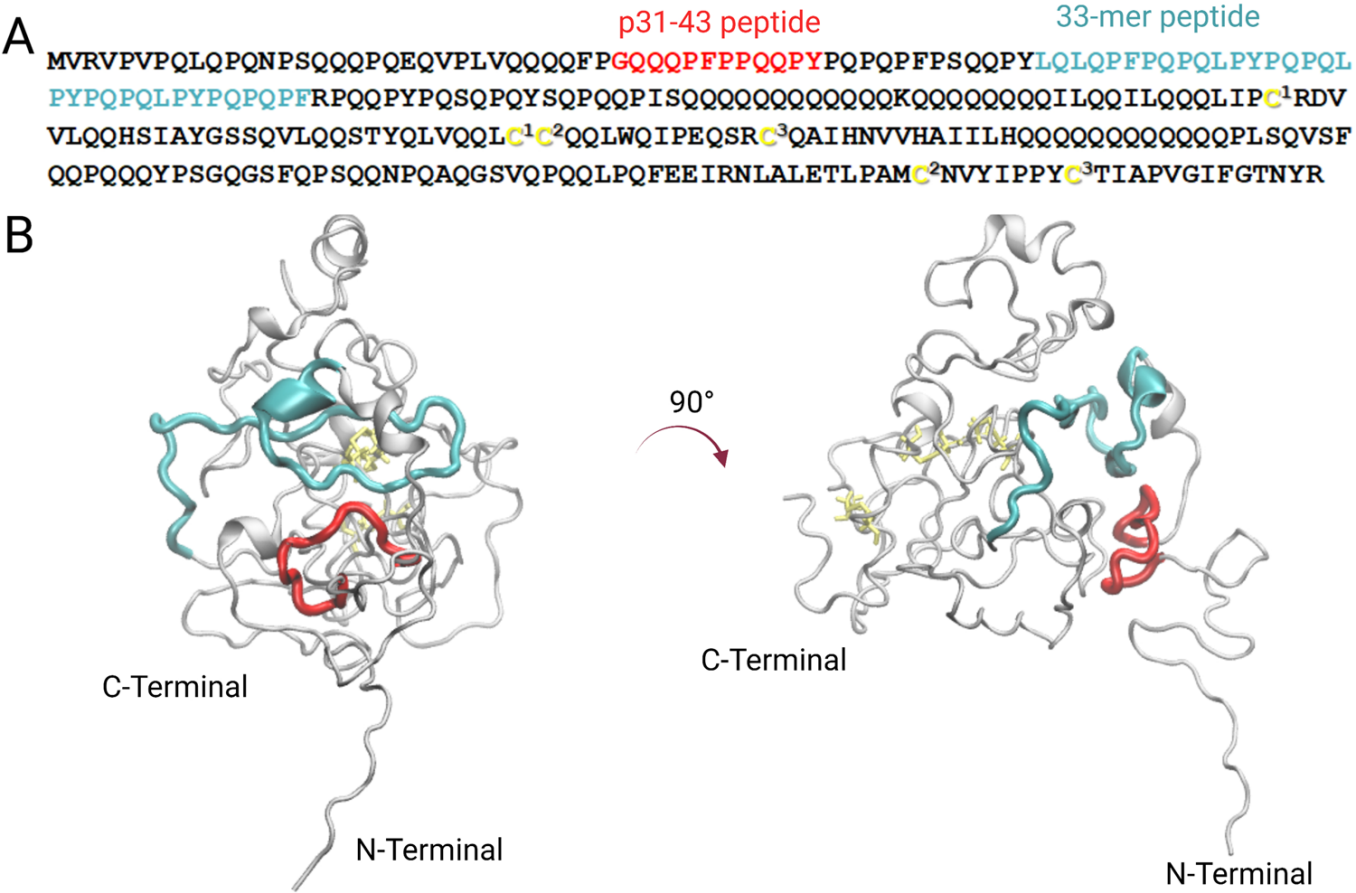
Structural analysis α-2-gliadin of *Triticum aestivum* where the corresponding regions of p31-43 (red) and 33-mer (cyan) are highlighted. The disulfide bonds are indicated in yellow. **A** Primary sequence of wheat α-2-gliadin (Q9M4L6). The six cysteines that form the three disulfide bonds are annotated in the following manner:^1^144–174, ^2^175–269 and ^3^277–187. **B** Tridimensional depictions of a model structure of α-2-gliadin after molecular dynamic simulation where disulfide bonds were incorporated. This image was adapted from Vazquez et al*.*, Int. J. Biol. Sci. 2021 under creative commons CCA (Vazquez et al. 2021)

Gliadin is composed of different isoforms that are classified due to their electrophoretic mobility in α (25–35 kDa), β (30–35 kDa), γ (35–40 kDa), and ω (55–75 kDa) (Osborne 1907; Quester et al. 2014). These proteins are also called prolamins due to their high content of proline and glutamine (Wieser 2007), as it could be observed in Fig. 1A. From a structural point of view, many studies have been done to obtain information about their secondary and tertiary structure; however, there is insufficient knowledge about their complete tridimensional structure. This could be explained by their low solubility in aqueous solutions and their high molecular weight, making it impossible to study these proteins by atomistic methods such as X-ray crystallography and nuclear magnetic resonance (NMR). However, low-resolution techniques such as circular dichroism (CD), fluorescence, infrared (IR), and Raman spectroscopy in combination with electron microscopy have been employed to evaluate gliadin conformation and self-assembly properties in solution (Tatham and Shewry 1985; McMaster et al. 2000; Blanch et al. 2003; Sato et al. 2015; Herrera et al. 2016, 2018b; Wouters et al. 2020). Recently, gliadin tridimensional structure was modeled by computational approaches to get insights in its structural features (Fig. 1B) (Vazquez et al. 2021). Moreover, it was determined that α/β and γ gliadins undergo liquid–liquid phase separation forming liquid droplets at different temperatures and concentrations (Boire et al. 2018; Sahli et al. 2019).

One of the major issues of gliadin in human nutrition is that it is not fully degraded by the human gastrointestinal enzymes, producing toxic or immunogenic peptides for susceptible individuals, as occur in celiac disease (Shan et al. 2005; Vazquez et al. 2021). These peptides are of particular interest because their diversity and concentration in food preparations could vary depending on the food matrix; however, the most toxic peptides remain after a simulated and in vivo gastric and pancreatic digestion (Prandi et al. 2014; Gutiérrez et al. 2017). To date, no efficient therapeutic approach has been developed to reduce or avoid their effects in CeD and the only treatment is a strict lifelong gluten-free diet (Scherf et al. 2020).

In recent years, attempts to get insights into structural properties of gliadin peptides are of general interest. Their conformation has an essential role in triggering the cellular effects described in gluten-related disorders. Manai et al*.* have recently shown by fluorescence and electron microscopy that pepsin-trypsin digest of gliadin can self-assemble in vitro and dysregulate autophagy processes in the Caco-2 cell line (Manai et al. 2018).

**Fig. 2:**
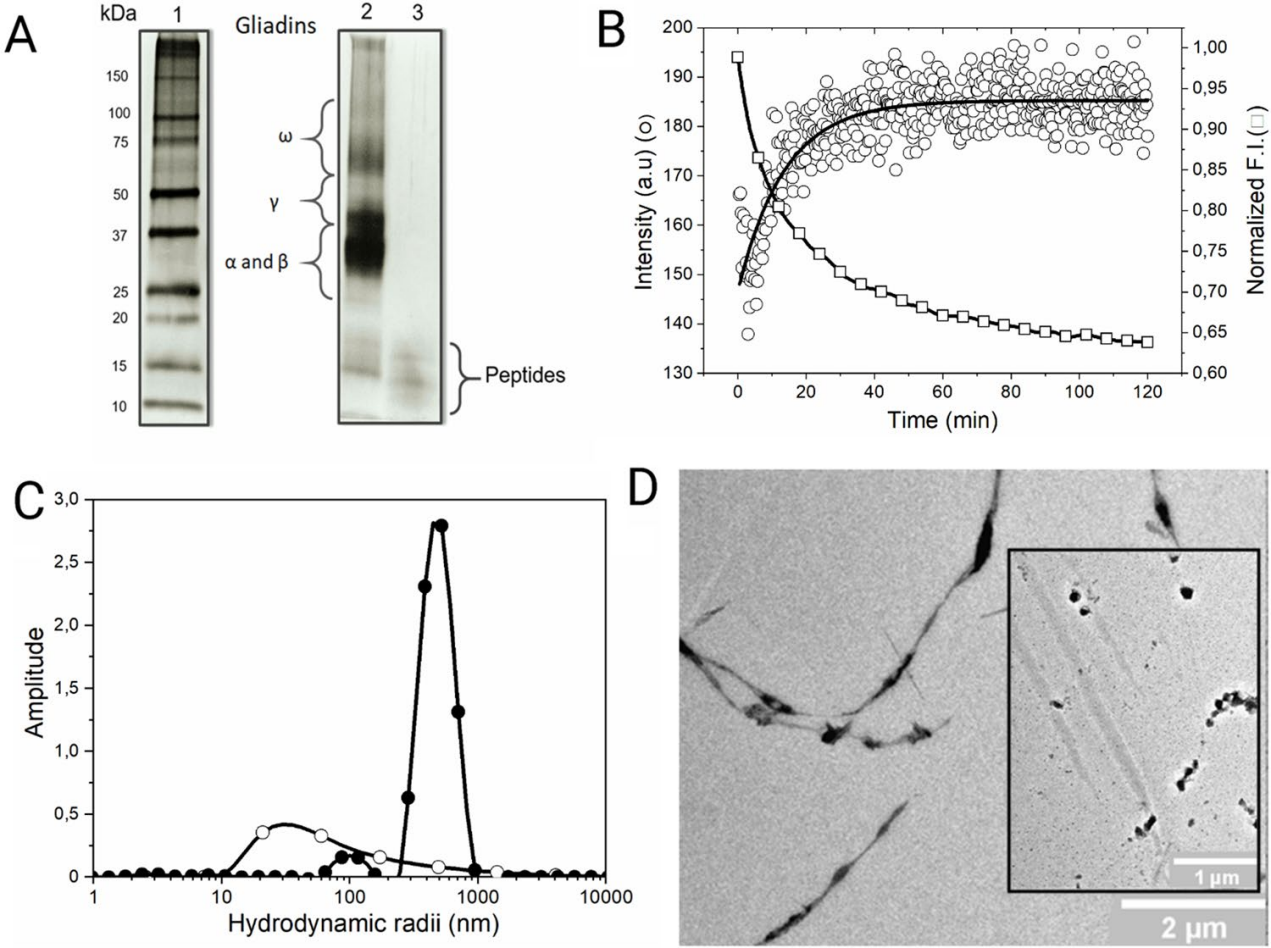
Biophysical and biochemical analysis of the structural and morphological properties of gliadin peptides obtained by pepsin digestion. **A** SDS-PAGE analysis showing the peptides that remain after gliadin pepsin degradation. **B** Kinetic analysis of gliadin degradation by pepsin using fluorescence and DLS. In the last, the formation of higher-order aggregates is observed during the proteolytical process. **C** DLS size distribution analysis of gliadin, (⚪) and gliadin peptides obtained after pepsin treatment (⚫). **D** Transmission electron microscopy of the pepsin digest of gliadin where fibrils and oligomers are observed. Adapted from Herrera et al*.*, Mol. Nutr. Food Res, 2021 under creative commons CCA (Herrera et al. 2021)

Recently, Dodero and collaborators have studied the structural and colloidal properties of a pepsin digest of gliadin. In this work, it has been observed that the proteolytical resistant gliadin peptides self-assemble during pepsin degradation (Fig. 2). When the mixture was analyzed by dynamic light scattering (DLS), the formed peptide structures have a higher hydrodynamic radius than their precursor gliadin (Fig. 2B and C) (Herrera et al. 2021). Also, the gliadin pepsin digest has a surfactant behavior in the air–water interface. Transmission electron microscopy (TEM) (Fig. 2D) and scanning electron microscopy (SEM) experiments show that these peptides form large structures as oligomers and fibrils. These structures presented an amyloid-like nature which was determined by the fluorescence emission of probes such as thioflavin T (ThT) and a BODIPY-based probe. The secondary structure analysis of this complex mixture revealed that the gliadin peptides present a higher content of parallel, anti-parallel β-sheets, turns, and less α-helix structure than gliadin. The presence of such nanostructures has been shown to induce the overexpression of several genes related to inflammation, such as the interleukin-8 and the chemokine receptor 3 (CXCL-8/CXCR3) axis in the Caco-2 cell line. This behavior is not observed when the cells are treated with 33-mer peptide, one of the known CeD implicated peptides, showing the importance of the mixture of peptides in the recapitulation of gluten-related diseases (Herrera et al. 2021). Recently, Feng et al*.* have isolated small cationic gliadin peptidic nanoparticles after digestion using ultracentrifugation. Fluorescence spectroscopy using the ThT probe enable the detection of these particles that self-assemble in solution in a concentration-dependent manner with a structural transition towards β-sheet structure above their critical aggregation concentration. The authors showed that these nanoparticles penetrate the mucus layer in Caco-2/HT29-MTX co-cultures. It was found that the nanoparticles bind to DPPC/DOPE vesicles employing isothermal titration calorimetry (ITC). This was also confirmed by the change of the surface pressure on DPPC/DOPE Langmuir monolayers. These observations suggest that these peptides could have a role in developing inflammation in the gut (Feng et al. 2021). Considering these last results by different research groups, it is possible to propose that evaluating the structure and activity of gliadin peptides as a whole system could help comprehend the complex molecular causes that drive gliadin-related disorders. In the meantime, analyzing disease-related peptides, as the 33-mer and the p31-43 (Fig. 1), is a successful strategy to evaluate the structural complexity of gliadin-digested peptides These fragments remain after gliadin digestion and they have high content glutamine (Q) and proline (P) in their primary structure. This indicates that they could adopt a polyproline II conformation (PPII), which is highly abundant in P-rich peptides (Adzhubei et al. 2013).

## The immunodominant 33-mer gliadin peptide self-assembles into oligomers and fibrils that activate an innate immune response in macrophages by Toll-like receptors (TLRs)

The 33-mer gliadin peptide (LQLQPF(PQPQLPY)_3_PQPQPF) has been recognized as the immunomodulator peptide that remains after α-2 gliadin proteolysis (Shan et al. 2002; Gutiérrez et al. 2017) and showing to be predominant in several wheat cultivars (Schalk et al. 2017). Once this peptide is in the gut mucosa, it can translocate from the lumen to the lamina propria, especially in celiac disease patients. It is proposed that endocytosis might be the most important mechanism of translocation; however, no specific receptor has been identified (Schumann et al. 2008; Ménard et al. 2012). On the other hand, it has been demonstrated that transglutaminase-2 (TG-2) binds to the 33-mer peptide and deamidate three glutamine residues of this peptide. Both peptides also showed that after being processed by the antigen presentation cells, they bind to human leukocyte antigen (HLA) type DQ2; however, the deamidated one presented a higher affinity towards this molecule (Qiao et al. 2004). Interestingly, the 33-mer peptide accumulates in the pancreas in a diabetes mouse model and closes K/ATP channels inducing insulin secretion in pancreatic model lines (Dall et al. 2013; Bruun et al. 2016). These results showed the importance of 33-mer peptide in developing multiorgan dysfunction and pathological immune responses beyond gluten-related disorders (López-Casado et al. 2018).

**Fig. 3:**
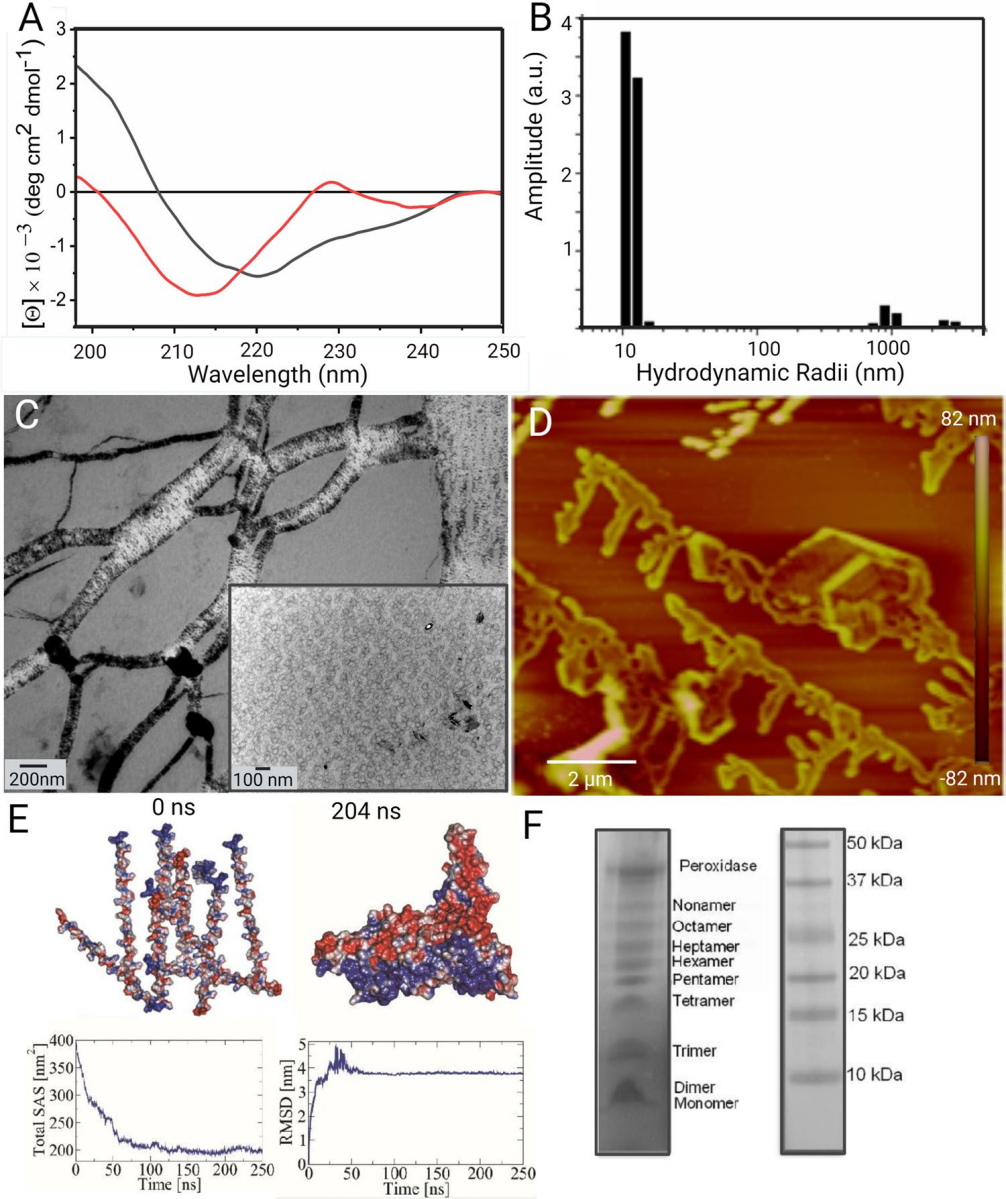
Conformational analysis of the 33-mer gliadin peptide by different biophysical and biochemical tools. **A** CD analysis at 600 µM at – 5 °C (red line) and 37 °C (dark line) adapted from Herrera et al., Biopolymers, 2014. **B** DLS indicating two main populations in solution (600 µM). Adapted from Herrera et al., Soft Matter, 2015. **C** TEM image showing the presence of fibrils and small−oligomers (600 µM) adapted from Herrera et al., Biopolymers, 2014. **D** AFM image at 600 µM showing the presence of the peptide quaternary structures. Adapted from Herrera et al., Soft Matter, 2015. **E** Molecular dynamic simulation of ten monomers in a solvated box depicted with electrostatic potential, showing the initial state of the dynamic and a representative structure of the oligomer obtained at 204 ns. Also presented are the total solvent accessible surface area (SAS) and the root-mean-square deviation (RMSD) using the Cα atoms over the simulation time compared to the initial structure. Adapted from Amundarain et al., Phys Chem Chem Phys,2019. **F** SDS-PAGE analysis of the peptide species after dityrosine cross-linking. The left line corresponds to a 50-µM sample treated with horseradish peroxidase and shows the presence of different oligomers, from the monomer to nonamers. The right line is the molecular weight marker. Adapted from Amundarain et al., PhysChemChemPhys, 2019

From a biophysical point of view, the first studies to assess the conformation of 33-mer were done by CD, showing that this peptide adopts a polyproline (II) PPII structure. An analysis by CD and NMR of the small PQPQLPY sequence, repeated three times in the 33-mer peptide, confirmed these structural observations (Parrot et al. 2002). Together with our collaborators, we performed a detailed analysis of the biophysical properties of the 33-mer peptide, showing that the peptide presents a PPII structure in equilibrium with other conformations depending on the peptide concentration by employing the complementary CD and attenuated total reflectance/Fourier transform infrared spectroscopy (ATR-FTIR) (Herrera et al. 2014, 2015). These studies demonstrated that at concentrations below 197 µM, this gliadin fragment presents a random conformation in equilibrium with a PPII structure. At higher concentrations, such as 600 µM, there is an equilibrium from PPII towards a β-parallel structure, which could be detected when the temperature is changed from 5 to 37 °C (Fig. 3A). By CD, these observations accompanied a hypochromic displacement of the signal, suggesting a self-assembly process. The last was confirmed by TEM and SEM analysis, which showed the formation of oligomers and fibrils (Fig. 3C). The fibrillar-like structures showed to cover a high degree of the surface and are interconnected with each other (Herrera et al. 2014). DLS experiments were carried out at different peptide concentrations, showing three populations in solution at all concentrations. Higher concentrations presented larger structures in equilibrium with smaller ones, suggesting a complex oligomeric equilibrium at the tested concentration (Fig. 2B). We also used Atomic Force Microscopy (AFM) due to its methodological advantages to evaluate the shape and sizes of the structures present in native-like conditions (Herrera et al. 2015). An analysis carried out in a concentration-dependent manner showed that low concentrations, such as 6 µM, the peptide assembly into oligomers and clusters. Then, increasing the concentration to 60 µM showed the formation of oligomers and planar structures resembling plaques and filaments. At 600 µM, linear interconnected filaments and plaques surrounded by spherical oligomers (5.1 ± 1.5 nm average height) structures were observed. These linear structures present a quaternary fractal nature, similar to those previously observed by TEM (Herrera et al. 2015), and these ensembles could be described by diffusion-limited aggregation (Fig. 3D).

Another microscopic tool used to evaluate 33-mer gliadin peptide was helium ion microscopy (HIM), which gave detailed morphological images without needing to coat the sample (Herrera et al. 2018a, b). This technique showed the association of spherical- and square-like oligomers into rod-like ones, which then organize into fibrillar structures. These larger structures have been shown to be the ones that induce the expression of NF-κβ via Toll like receptors (TLR)-2 and -4 accompanied with the induction of the pro-inflammatory related molecules C-X-C motif chemokine ligand 10 (IP-10/CXCL10) and tumor necrosis factor-α(TNF-α) (Herrera et al. 2018a, b). These last results strongly suggest the interplay between the 33-mer gliadin structure and inherent self-assembly capability with its inflammatory response.

To get insights into the molecular mechanism and driving forces of the first steps of 33-mer gliadin peptide oligomerization, Amundarain et al*.* have performed an exhaustive molecular dynamic simulation analysis (Amundarain et al. 2019). The authors evaluated the conformational properties of the monomer and the formation of different oligomers such as dimers, trimers, and decamers, showing the high tendency of this peptide to self-organize and been in a conformational equilibrium between PPII and β-structures. These observations could be explained by the high proline and glutamine content and the partial charge distribution. In this peptide, glutamines have been shown to favor H-bonds between the monomers, triggering the formation of oligomers (Fig. 3E). Besides, a di-tyrosine cross-linking assay was conducted to get experimental proof on the first steps of oligomerization. After this reaction, polyacrylamide gel electrophoresis analysis showed different oligomeric forms from dimers to at least a nonamers confirming the computational analysis (Fig. 3F) (Amundarain et al. 2019).

## The p31-43 peptide has a polyproline structure and self-assembles into oligomers inducing NLRP3 inflammasome activation in a mouse model

The 31–43 peptide (LGQQQPFPPQQPY, p31-43) sequence has been detected mainly in the Q9ZP09 isoform from α-gliadin, been part of the resistant proteolytical peptide named as 25-mer. p31-43 has shown toxic effects in the gut mucosa, such as the induction of interleukin-15 (IL-15) (Maiuri et al. 2003; Mamone et al. 2007). However, this peptide does not bind to the HLA-DQ molecules, which indicates that it cannot elicit an adaptive immune response. Instead, this peptide activates the innate immune pathways in vivo in an interferon (IFN)-dependent manner, showing an increment of intraepithelial migration of CD8 + T cells and enterocyte apoptosis, recapitulating the histological damages observed in CeD (Araya et al. 2016). Furthermore, this fragment translocates into the cell with an unknown mechanism and induces a cellular endocytosis dysfunction, inhibiting endosomal maturation (Barone et al. 2010; Nanayakkara et al. 2018). To date, no receptor has been identified to promote its internalization. Recently, it was observed that the peptide could interact with the cystic fibrosis transmembrane conductance regulator (CFTR) anion channel, inhibiting its ATPase activity and activating TG2. These activities led to cellular stress, cytoskeleton disassembly, and inflammasome activation (Villella et al. 2019).

A first molecular analysis showed that the intrinsic fluorescence emission of the tyrosine residue changes in the presence of sodium dodecyl sulfate (SDS) micelles, which can act as a simple model membrane, suggesting that this peptide could diffuse in a biomembrane-like environment (Vilasi et al. 2010). The group of Chirdo and collaborators (Gómez Castro et al*.* 2019) studied the secondary structure of this peptide by CD, observing that it presents a PPII structure and by employing TEM showed that it could self-assemble in oligomers. Also, the authors showed in this article by molecular dynamic simulations the capacity of this peptide to self-assemble into oligomers with a high content of PPII structure. By combining *in cell* and in vivo experiments, the authors demonstrate that p31-43 induces NLRP3 inflammasome activation, leading to caspase-1-dependent intestinal inflammation (Gómez Castro et al*.* 2019). Then, the same group has made a detailed biophysical analysis of this peptide using spectroscopic tools and AFM (Herrera et al*.* 2020). These structural results were confirmed by the group of Barone and co-workers, using NMR spectroscopy which showed that the peptide monomer in the presence of SDS surfactant adopts a PPII in an *all trans* conformation that it is in equilibrium with a *cis* conformation (Calvanese et al. 2019). Also, the authors showed using computational docking experiments that this peptide is a weak ligand to HLA-DQ and T receptor, indicating that these structural features could play a role in why this peptide does not elicit an adaptative immune response (Calvanese et al. 2019; Falcigno et al. 2020).

In additions to these results, Herrera et al*.* evaluated p31-43 using various biophysical tools such as DLS, which showed that the peptide at 125 µM has two main oligomerization states in solution, one around 100 nm, and other population around 500 nm. By fluorescence spectroscopy, it has been observed that this peptide can bind to BODIPY-based probe that senses oligomers (Herrera et al*.* 2020). Then, the study of the peptide structure and self-assembly at different concentrations by CD revealed a conformational equilibrium between PPII to more folded structures such as β-turns and β-sheet-like structures above 200 µM. Finally, the AFM analysis over time at the peptide concentration of 10 µM and 50 µM showed that its self-assembly depends on the time. At the lowest concentration, the peptide forms mainly spherical oligomers that coalesce over time. At the highest one, oligomers interact with each other, forming large linear arrangements (Herrera et al*.* 2020). Barrera et al*.* have recently dissected the role of glutamines in the self-assembly of this peptide, showing that this process could be divided into three phases that ended in the formation of a 50-mer aggregate with an increase of the β-extended structure (Barrera et al. 2021).

## Conclusions

In summary, we have discussed the recent results obtained in the study of gliadin proteolytical resistant peptides. Gliadin peptides remain partially non-digested in the gut mucosa of healthy individuals and celiac patients, eventually inducing cellular damage in the small intestine in susceptible individuals. The exacerbation of the immune response is only controlled by adopting a gluten-free diet. It has become of key importance to understand their molecular properties and how they affect predispose individuals. All the peptides mentioned have been shown to adopt a PPII structure and tend to self-assemble with a conformational transition towards β structures. In vitro, these morphological and structural features are accompanied by cellular impairment and increase of the levels pro-inflammation markers that resemble the biochemical and pathological responses observed in celiac disease and gluten-sensitive patients.
